# Inactivation of Lipopolysaccharide-Biosynthesizing Genes Altered Lipids Composition and Intensity in *Cronobacter sakazakii*

**DOI:** 10.3390/pathogens13121035

**Published:** 2024-11-23

**Authors:** Xiaoqing Hu, Xi Yang, Shuyan Wu, Xuan Lu, Yuan Ma, Ziyi Tang, Xiaoyuan Wang, Xiaodong Huang, Shuiping Wang

**Affiliations:** 1 State Key Laboratory of Food Science and Resources, Jiangnan University, Wuxi 214122, China; 6200201050@stu.jiangnan.edu.cn (X.Y.); xwang@jiangnan.edu.cn (X.W.); 2 Biotechnology School, Jiangnan University, Wuxi 214122, China; 6230201028@stu.jiangnan.edu.cn (X.L.); 6230209055@stu.jiangnan.edu.cn (Y.M.); 6230209017@stu.jiangnan.edu.cn (Z.T.); 3 AgResearch Ltd., Hopkirk Research Institute, Palmerston North 4442, New Zealand; shuyanwu@agresearch.co.nz; 4 Guangzhou YueHui Cosmetics Co., Ltd., Guangzhou 510440, China; shym001@mfsj1908.com; 5 Guangzhou CnFerment Biotechnology Co., Ltd., Guangzhou 510440, China; shym060@mfsj1908.com

**Keywords:** lipopolysaccharide, *Cronobacter sakazakii*, lipidomics, unsaturated lipid, lipid intensity

## Abstract

Gram-negative bacteria possess an asymmetric outer membrane, where the outer leaflet consists of LPSs and the inner leaflet comprises phospholipids. *Cronobacter sakazakii*, an opportunistic milk-borne pathogen that causes severe neonatal meningitis and bacteremia, displays diverse lipopolysaccharide (LPS) structures. As a barrier of the bacterial cell, LPSs likely influenced *C. sakazakii* resistance to environment stresses; however, there are no research reports on this aspect, hindering the development of novel bactericidal strategies overcoming the pathogen’s resilience. In the present study, therefore, *C. sakazakii* BAA894 and two LPS mutants (Δ*lpxM* and Δ*waaC*) were employed to investigate its influences. The Δ*waaC* mutant showed lower resistance to acidic, alkali, oxidative, and osmotic stresses compared to the wild-type strain BAA894, and the Δ*lpxM* mutant exhibited lower desiccation resistance but higher osmotic resistance. To uncover potential reasons for these differences, comparative lipidomics was conducted. The results showed that compared to BAA894, both mutants showed drastic changes in lipid quantity, and many changed lipids were unsaturated. Additionally, eleven lipid classes exhibited significant variation in the relative content. In particular, the polyunsaturated TGs with double bonds at 5, 7, 12, and 14 displayed significant variation between the wild type and two mutants. Our study is the first to reveal that the changes in the LPS structure of *C. sakazakii* resulted in altered lipid profiles and intensities, which may be a critical biochemical basis for bacterial resistance to harsh stresses.

## 1. Introduction

*Cronobacter sakazakii* is a prevalent pathogenic bacteria responsible for a range of diseases, posing threats to both human health and economy. As a Gram-negative facultative anaerobic rod-shaped enterobacteria widespread in the environment, *C. sakazakii* frequently contaminates powdered infant formula. Infection with *C. sakazakii* is rare; however, it can be deadly for young infants, with a mortality rate of 4–80% [1]. Similarly to other enterobacteria, *C. sakazakii* showed serological heterogeneity due to the diversity in lipopolysaccharide (LPS) structure. LPSs are a major component of the outer leaflet of the outer membrane in Gram-negative bacteria and are often a crucial factor of their virulence. Through modifying its LPS structure, *C. sakazakii* could evade recognition of the host immune system [2]. These results indicate that LPSs play a key role in *C. sakazakii* pathogenicity.

The complete structure of LPSs comprises three regions: hydrophilic O-antigen, core oligosaccharide, and hydrophobic lipid A. The lipid A and inner core region displayed the highest degree of structural conservation while the O-antigen showed the greatest variability. The O-antigen was the most variable constituent, and its variation provided the basis for Gram-negative bacteria serotyping. Based on MboII restriction fragment length polymorphism (RFLP) patterns, *C. sakazakii* showed three RFLP clusters of the O-antigen gene, previously named as serotypes O1, O2, and O3 [3]. On the other hand, the O-antigen may be absent. For instance, some pathogenic bacteria possess short-chain LPSs, also referred to as lipooligosaccharides, which lack the O-antigen component.

The LPS core and lipid A together help maintain the integrity of the outer membrane [4].The core oligosaccharide of LPSs, which connects the O-antigen and lipid A, is a fundamental component found in all LPS structures known in nature [4]. The oligosaccharide core is further divided into inner and outer regions. The inner core is formed by at least one Kdo and two units of L-glycero-D-manno-heptose (Hep) [5], and is well conserved and important for the stability of the outer membrane [6]. During the biosynthesis of the inner core, WaaC (encoded by *waaC*) is a crucial enzyme, responsible for adding the first heptose to the inner 3-deoxy-d-manno-oct-2-ulosonic acid (Kdo) of Kdo2-lipid A. In our previous study [7], the gene *ESA_04107* (*waaC*) was identified in *C. sakazakii*, and *C. sakazakii* BAA-894 Δ*waaC* showed increased biofilm-forming ability compared to BAA894, due to its stronger cell surface hydrophobicity auto-aggregation rate. Another work showed that a mutant inner core gene in *E. coli* increased electro-transformation efficiency [8].

As another essential component of LPSs, lipid A functions as the hydrophobic anchor of LPSs embedded within the outer membrane of bacteria. Similarly to that in *E. coli*, lipid A in *C. sakazakii* comprises four 3-hydroxyacyl chains at the 2-, 3-, 2′-, and 3′-positions, respectively, with two additional acyl chains linked to the 2′- and 3′-hydroxyacyl chains, forming acyloxyacyl moieties [9]. In our previous report [10], the gene *ESA01386* in *C. sakazakii* BAA-894 was identified as the homolog of *lpxM* in *E. coli*, and the encoded acyltransferase LpxM is responsible for transferring a C14:0 secondary acyl chain to the 3′-position of lipid A. Consequently, *C. sakazakii* BAA-894Δ*lpxM* produced a modified lipid A with four 3-hydroxyacyl chains and a single acyl chain, one less than that in the wild type, and regarded as a classical mutant of lipid A [10].

LPSs serve as a permeability barrier of bacterial cells. In our previous study, the effects of various LPS mutants of *E. coli* W3110 on outer membrane permeability were investigated. The results showed that eight LPS mutants (including Δ*waaC*, Δ*lpxM,* and six other variants) exhibited higher outer membrane permeability compared to wild-type W3110. However, to the best of our knowledge, the impacts of LPS mutants on the bacterial resistance to environmental stresses (acid, alkali, oxidative, osmotic, and desiccation stresses) have not been systematically investigated.

*C. sakazakii* can survive in a variety of foods, especially powdered infant formula, and possesses high persistence during food processing and storage, which may be attributed to the strong resistance of *Cronobacter* spp. to environment stresses such as osmotic pressure, low pH, heat, oxidation, and desiccation [11]. Specifically, *C. sakazakii* is able to survive for approximately two years in powdered infant formulas [12]. Numerous factors contribute to the survival of *Cronobacter* spp. in harsh environments, including specific genes, regulatory systems, and the formation of biofilms [11]. These mechanisms collectively enable Cronobacter spp. to withstand adverse conditions and persist in challenging environments. It was of significance to identify the factors contributing to the high resistance of *C. sakazakii* to these environmental stresses. For example, the maltodextrin-binding protein encoded by *ESA_03421* was reported as the key gene involved in the thermotolerance of *C. sakazakii* during desiccation through a comparative proteomics analysis [13]. Other influencing factors were also reported recently, such as but not limited to flagellar gene *flgK* controlling desiccation resistance [14], *dnaK* gene regulating acid resistance [15], and sigma factor RpoS’s involvement in environmental stress resistance [16]. In our previous report [17], the varied composition of carotenoids also significantly affected the thermotolerance of *C. sakazakii* by altering the lipid profile and intensity and changing the cell membrane properties. However, there is currently little literature available on the role of LPSs in bacterial tolerance to environmental stresses (such as osmotic pressure, low pH, heat, oxidation, and desiccation) that are commonly encountered in dairy processing. In the present study, two critical genes involved in LPS biosynthesis and modification in *C. sakazakii* BAA-894, namely *lpxM* and *waaC*, were inactivated, respectively. The impact of these gene knockouts on the bacterium’s resistance to various environmental stresses was then evaluated.

How LPS modification influences bacterial resistance to chemical and physical stress remains unclear. Previous reports suggest an interaction exists between two major components of the outer membrane, LPSs and phospholipids [18]. Thus, changes in LPS structure could possibly alter lipid profile and content. On the other hand, there is a close relationship between bacterial resistance and cell membrane stability, which is strictly dependent on biochemical components such as the lipid profile [11]. Based on these findings, it was of great significance to explore the possible changes in the lipidomics of LPS mutants. As a subcategory of metabolomics, lipidomics focuses on the large-scale identification and quantification of various lipids within biological systems and has been important in understanding the roles of lipids in cellular functions. With the improvements in chromatographic separation and mass spectrometry in recent years, lipidomics has experienced significant progress [19,20]. In a recent study, we employed comparative lipidomics for the first time to reveal the global lipids in *C. sakazakii*, and their changes after inactivation of carotenogenic-biosynthesizing genes [17]. The lipidomics analysis revealed that alterations in carotenoid composition in *C. sakazakii* significantly impacted lipid composition and intensity, which could be a key factor influencing the properties of the cell membrane and stress resistance [17]. Building upon this, comparative lipidomics was also utilized in the current study to examine the detailed variations in lipid composition and intensity in LPS mutants. The present work aims to enhance the understanding of the physiological roles of LPS modification in *C. sakazakii* adaption to environmental stresses.

## 2. Materials and Methods

### 2.1. Strains and Growth Conditions

The two LPS mutants, Δ*waaC* [7] and Δ*lpxM* [10], of *C. sakazakii* BAA894 (ATCC, Murfreesboro, TN, USA), were constructed in our previous study. All strains were grown at 30 °C in Luria–Bertani (LB) medium, composed of 5 g yeast extract (Oxoid Ltd., Hampshire, UK), 10 g tryptone (Oxoid Ltd., Hampshire, UK), and 10 g NaCl (Sinopharm Chemical Reagent Co., Ltd., Beijing, China) per 1 L (Table 1).

### 2.2. Inactivation Experiments Under Environmental Stress

The resistance of wild-type and mutant strains to various environmental stresses (acidic, osmotic, and oxidative) was assessed following the method described by Sedkova et al. [21], with minor modifications as detailed below. Firstly, bacterial cells in the logarithmic growth phase were harvested and suspended in LB medium to an OD_600_ of 1.0. The bacterial suspensions were then subjected to the following stress conditions: (1) acid stress, pH was adjusted to 3 using HCl (Sinopharm Chemical Reagent Co., Ltd., Beijing, China) solution (10 M); (2) alkali stress, pH was raised to 11 using NaOH (Sinopharm Chemical Reagent Co., Ltd., Beijing, China) solution (4 M); (3) oxidative stress, 30 mM of 30% H_2_O_2_ (Sinopharm Chemical Reagent Co., Ltd., Beijing, China) was added; and (4) osmotic stress, 10% NaCl was added. After exposure to these conditions for 30 min (acidic, alkali, and oxidative stresses) or for 90 min (osmotic stress), the cell suspensions were serially diluted in 0.85% NaCl solution and plated on LB medium to count the viable cells. The inactivation rate was calculated by comparing the bacterial colony counts between unstressed and stressed cells. Additionally, desiccation experiments were conducted following the method reported by Chen et al. [22].

### 2.3. Lipid Extraction and Analysis

Lipids were extracted using a modified Folch protocol with minor modification as follows [23]. Briefly, stationary-phase cells were centrifuged, washed with PBS (Sinopharm Chemical Reagent Co., Ltd., Beijing, China), and resuspended to an OD_600_ of 4.0. One mL of each suspension was transferred to a 1.5 mL microcentrifuge tube, and then centrifuged. The resultant pellet was frozen in liquid nitrogen for 10 min, then resuspended in 300 µL of chloroform/methanol (Sinopharm Chemical Reagent Co., Ltd., Beijing, China) (2:1 *v*/*v*) with 100 µL of glass beads (Sigma-Aldrich, MO, USA) at room temperature, followed by vortexing for 15 min. Later, the mixture was centrifuged at 2000 rpm for 15 min, and the organic phase was carefully collected using a pipette. A second lipid extraction was performed on the aqueous phase using 300 µL of chloroform/methanol (2:1 *v*/*v*), and the two organic phases obtained were combined. Finally, the combined extract was evaporated under nitrogen and dissolved in 200 µL of chloroform/methanol (2:1 *v*/*v*) for UPLC-MS (UHPLC Ultimate 3000, Thermo Fisher Scientific, Waltham, MA, USA; Q Exactive Plus Orbitrap, Thermo Scientific, Waltham, MA, USA) analysis.

### 2.4. Data Analysis

Statistical analysis was performed using GraphPad Prism 4 (GraphPad Software Inc., San Diego, CA, USA). Bacterial colony counts were expressed by log CFU/mL, with means and standard deviations calculated accordingly. Multiple comparisons were conducted by one-way ANOVA, followed by Tukey’s HSD (honestly significant difference) post hoc test. For lipid analysis, raw data were analyzed using Lipid Search Software Version 4.1 (Thermo Fisher Scientific, Waltham, MA, USA) to identify lipid species.

## 3. Results

### 3.1. Effect of LPS Mutant on Bacterial Resistance to Environmental Stresses

**Figure 1:**
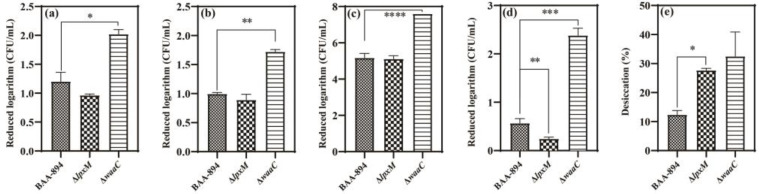
Inactivation kinetics of the wild type and two mutants under the stress conditions of HCl (**a**), NaOH (**b**), H_2_O_2_ (**c**), NaCl (**d**), and desiccation (**e**). ‘*’ for *p* < 0.05, ‘**’ for *p* < 0.01, ‘***’ for *p* < 0.001, and ‘****’ for *p* < 0.0001. If there is no mark between the columns, it indicates *p* > 0.05.

The bacterial resistance to harsh stresses is considered as the capacity for bacteria to adapt and persist under harsh environmental conditions, and is closely dependent on the characteristics of the cell membrane [24,25]. As shown in Figure 1a, under acidic stress, there was no significant difference in killing efficiencies between BAA894 and Δ*lpxM*. However, Δ*waaC* showed a reduction in viable cells of 1.5 log CFU/mL, indicating lower resistance to acid stress compared to BAA894 (*p* = 0.022). Similarly, under alkali stress (Figure 1b) and oxidative stress (Figure 1c), Δ*lpxM* did not show a significant change in resistance (*p* = 0.17). However, Δ*waaC* exhibited greater sensitivities to NaOH (*p* = 0.020) and to H_2_O_2_ (*p* = 0.000039).

The two other stress resistance assays revealed distinct changes in Δ*lpxM*. Surprisingly, under osmotic stress, a significantly lower bactericidal efficiency was seen in Δ*lpxM* (Figure 1d) (*p* = 0.0041), suggesting the deficiency of an acyl chain in lipid A enhanced *C. sakazakii* resistance to NaCl-induced osmotic stress. As shown in Figure 1e, after desiccation treatment, inactivation was significantly high in the Δ*lpxM* group, with a killing rate that was 25% higher than that of the BAA894 group (*p* = 0.031). This indicated that the removal of an acyl chain at the 3′-position of lipid A could compromise bacterial resistance to desiccation. In addition, Δ*waaC* showed significantly lower resistance to osmotic stress compared to BAA894 (*p* = 0.00039) but had unchanged resistance to desiccation stress (*p* = 0.078). It should be noted that under all stress conditions except for desiccation, Δ*waaC* showed an additional reduction of at least 0.7 log CFU/mL compared to BAA894.

### 3.2. Variations in Lipid Profile in LPS Mutants

It is assumed that changes in LPS structure lead to significant alterations in bacterial resistance to stresses that are closely related to cell membrane components. To uncover the biochemical basis, a comparative lipidomics analysis of *C. sakazakii* was conducted. In *C. sakazakii* BAA-894, 577 lipid species were identified based on the head group, fatty acid tail length, and saturation.

**Figure 2:**
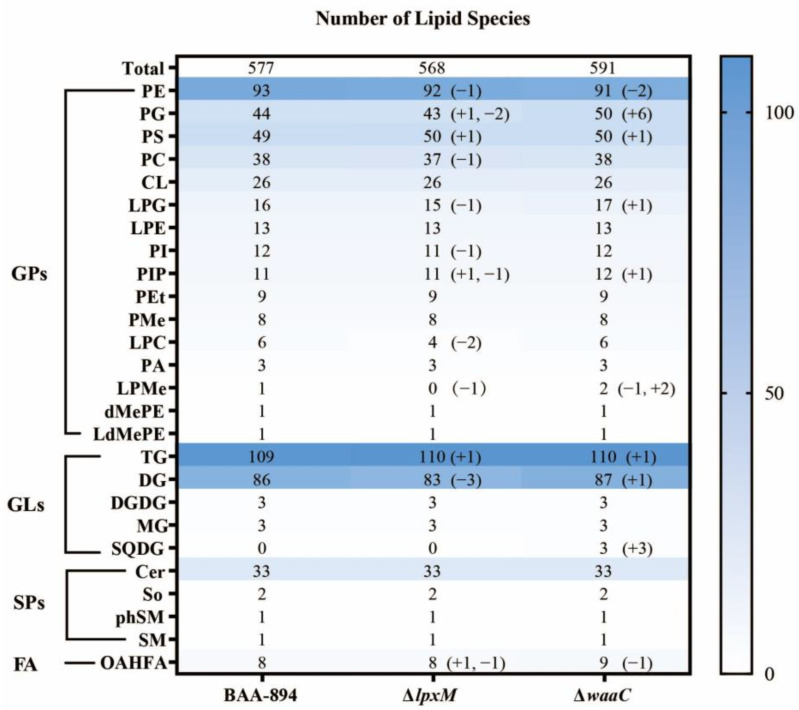
Changes in the number of total lipid species in all strains. If a bracket follows the numerical value, it indicates the detailed change in lipid species for the mutant compared to BAA894: ‘‘+’’ means the number increased and ‘‘−’’ means that it decreased.

These species belong to 26 lipid classes which can be further categorized into four groups as follows (Figure 2):

(1) Glycerophospholipid (GP) category, including cardiolipin (CL), phosphatidic acid (PA), phosphatidylcholine (PC), phosphatidylethanolamine (PE), phosphatidylglycerol (PG), phosphatidylinositol (PI, PIP), phosphatidylserine (PS), phosphatidyl ethanolamine (PMe), dimethylphosphatidyl ethanolamine (dMePE), lysophosphatidylglycerol (LPG), lysophosphatidylethanolamine (LPE), lysophosphatidylcholine (LPC), lysophosphatidylmethanol (LPMe), lysodimethylphosphatidylethanolamine (LdMePE), and phosphatidyl ethanol (PEt).
(2) Glycerolipids (GLs) including monoacylglycerol (MG), diradylglycerol (DG), triacylglycerol (TG), sulfoquinovosyldiacylglycerol (SQDG), and digalactosyldiacylglycerol (DGDG).
(3) Sphingolipids (SPs) comprising ceramide (Cer), sphingomyelin (SM), sphingomyelin (phytosphingosine) (phSM), and sphingosine (So).
(4) Fatty acyls (FAs) containing sole o-acyl-1-hydroxyl fatty acids (OAHFAs).

Compared to BAA894, the two mutants showed significant changes in lipid quantity. The total number of lipid species decreased to 568 species in Δ*lpxM* and increased to 591 species in Δ*waaC* (Figure 2), indicating the alterations in the LPS inner core region and lipid A changed the lipid composition. Interestingly, while there were both similarities and differences in the lipid changes between the two mutants, some lipids were affected in both. In particular, TG(26:5/18:1/23:6), an unsaturated TG with twelve double bonds, PS(18:1/23:6), an unsaturated PE with seven double bonds, PIP(32:2/16:0), an unsaturated PIP with two double bonds, and OAHFA(16:1/16:0), a monounsaturated FA, were observed in both mutants. Additionally, PE(18:1/22:0), a monounsaturated PE, was absent in both mutants.

Secondly, many of the varied lipids were unsaturated. In Δ*waaC*, three novel SQDGs [SQDG(16:0/21:2), SQDG(16:1/21:3), and SQDG(18:1/23:3)] and six novel PGs [PG(34:1/16:1), PG(29:5/18:1), PG(16:0/24:7), PG(27:4/18:1), PG(16:1/22:6), and PG(18:1/24:7)] were polyunsaturated. In Δ*lpxM*, most of the lipids that were absent were also unsaturated. For instance, two LPCs disappeared [LPC(18:2) and LPC(20:4)] that were polyunsaturated, and among three DGs that disappeared [DG(10:0e/14:1), DG(12:0p/14:0) and DG(12:0e/14:1)], two of them were monounsaturated.

### 3.3. Changes in Lipid Intensity in LPS Mutants

**Figure 3:**
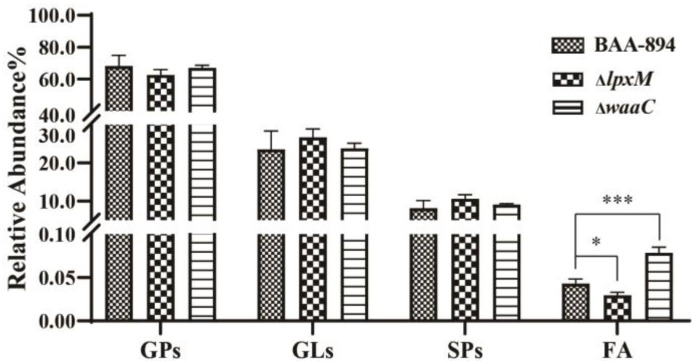
The relative abundance of four lipid groups in BAA-894 and two mutants. Error bars represent standard deviations from three samples. ‘*’ for *p* < 0.05, and ‘***’ for *p* < 0.001. If there is no mark between the columns, it indicates *p* > 0.05.

Not only was the profile of lipids affected but the relative intensity of global lipids was also influenced by truncation of the inner core polysaccharide (Δ*waaC*) and removal of the deacyl of lipid A (Δ*lpxM*) in LPS. Among the three major lipid categories (GPs, GLs, and SPs), no significant changes in the intensity of any category were detected (Figure 3), but twelve lipid classes exhibited significant variations. Additionally, the FA category highlighted differences in lipid content among the mutants.

**Figure 4:**
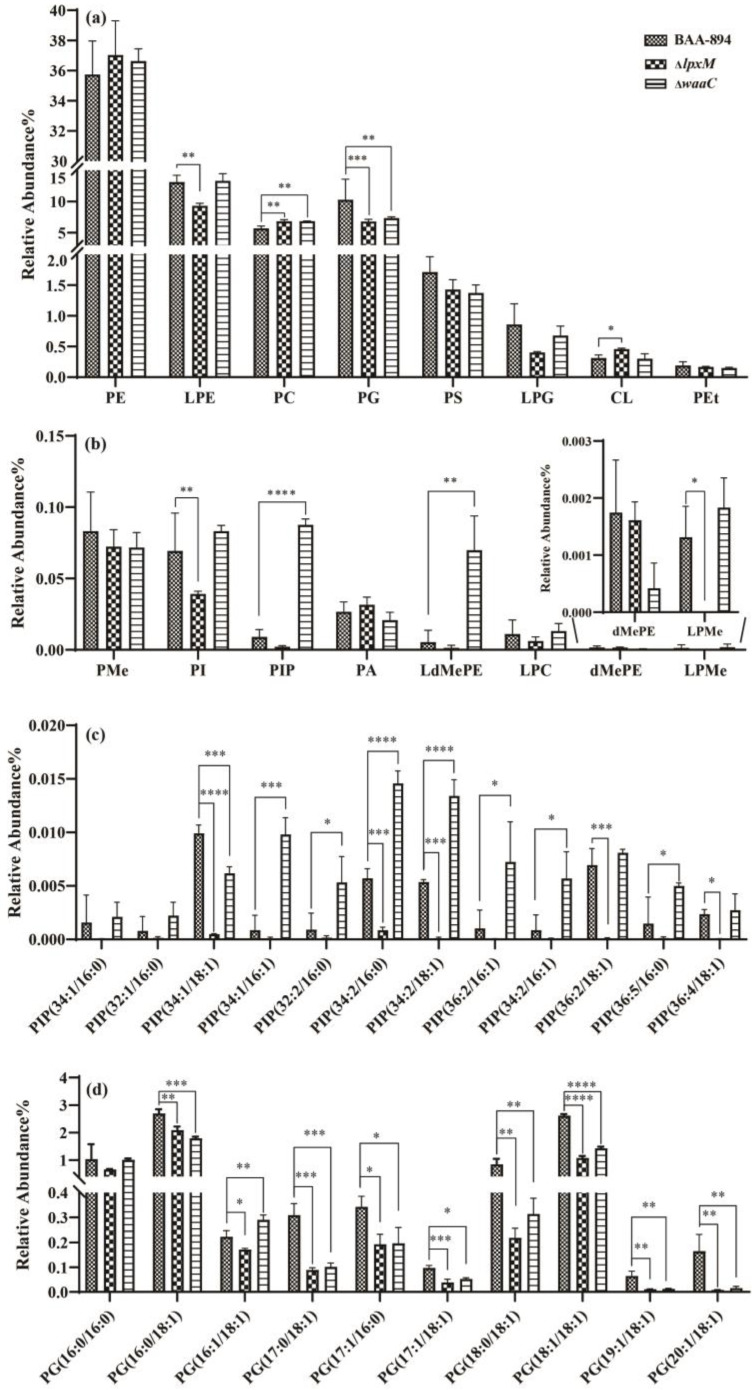
Abundances of glycerophospholipids (GPs). (**a**) Lipids with high abundance in GPs, including phosphatidylethanolamine (PE), lysophosphatidylethanolamine (LPE), phosphatidylcholine (PC), phosphatidylglycerol (PG), phosphatidylserine (PS), lysophosphatidylglycerol (LPG), cardiolipin (CL), and phosphatidyl ethanol (PEt); (**b**) lipids with low abundance in GPs, including phosphatidylinositol (PI, PIP), lysodimethylphosphatidylethanolamine (LdMePE), phosphatidyl ethanolamine (PMe), phosphatidic acid (PA), lysophosphatidylcholine (LPC), lysophosphatidylmethanol (LPMe), and dimethylphosphatidyl ethanolamine (dMePE); (**c**) relative lipid content of PIP; (**d**) the lipid relative content of Δ*lpxM* decreased the most obvious 10 kinds of PGs. Error bars indicate standard deviation. ‘*’ for *p* < 0.05, ‘**’ for *p* < 0.01, ‘***’ for *p* < 0.001, and ‘****’ for *p* < 0.0001. If there is no mark between the columns, it indicates *p* > 0.05.

In the GP category, eight lipid classes (LPE, PC, PG, CL, PI, PIP, LdMePE, and LPMe) showed significant changes in lipid intensity compared to BAA894. As shown in Figure 4, Δ*waaC* displayed increases of 20.16% in PC (*p* = 0.0026), 942.53% in PIP (*p* = 0.00000516), and 796.85% in LdMePE (*p* = 0.000055), respectively. Specifically, seven lipid species in PIP, including PIP(34:1/16:1), PIP(32:2/16:0), PIP(34:2/16:0), PIP(34:2/18:1), PIP(36:2/16:1), PIP(34:2/16:1), and PIP(36:5/16:0), showed increases of 1054.34%, 490.49%, 155.07%, 150.95%, 604.29%, 567.43%, and 239.08%, respectively, compared to BAA894. Additionally, Δ*lpxM* exhibited reductions of 28.86% in LPE (*p* = 0.009), 33.77% in PG (*p* =0.00052), 43.51% in PI (*p* = 0.0021), and 99.73% in LPMe (*p* = 0.0204), respectively (Figure 4). In particular, nine lipids in PGs [PG(16:0/18:1), PG(16:1/18:1), PG(17:0/18:1), PG(17:1/16:0), PG(17:1/18:1), PG(18:0/18:1), PG(18:1/18:1), PG(19:1/18:1), and PG(20:1/18:1)] exhibited significant reductions.

**Figure 5:**
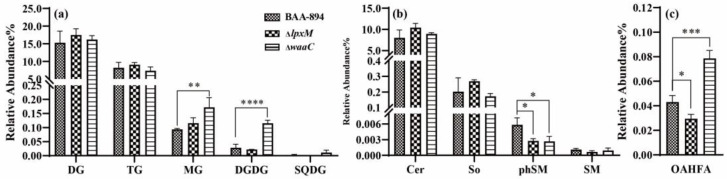
Abundances of representative lipid classes in (**a**) glycerolipids (GLs), (**b**) sphingolipids (SPs), and (**c**) fatty acyls (FAs). Error bars indicate standard deviation. ‘*’ for *p* < 0.05, ‘**’ for *p* < 0.01, ‘***’ for *p* < 0.001, and ‘****’ for *p* < 0.0001. If there is no mark between the columns, it indicates *p* > 0.05.

In the GL category (Figure 5a), both MG (*p* = 0.0096) and DGDG (*p* = 0.000061) were elevated in Δ*waaC* compared to BAA894. In the SP category (Figure 5b), phSM was decreased in both mutants (*p =* 0.0155 in Δ*lpxM* and 0.0137 in Δ*waaC*). In the FA category (Figure 5c), OAHFA was decreased in Δ*lpxM* (*p =* 0.035) but increased in Δ*waaC* (*p* = 0.0003). These results demonstrated the diverse variations in lipid profiles induced by changes in the LPS structure.

**Figure 6:**
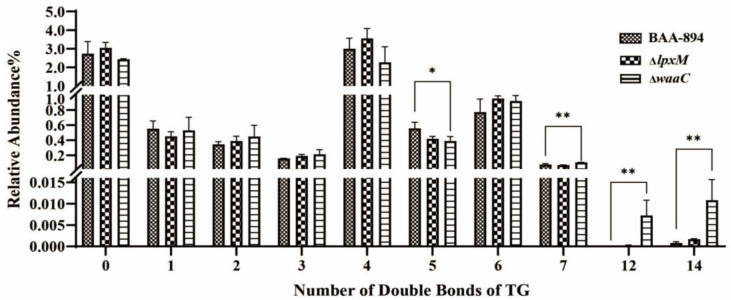
Abundance of unsaturated triacylglycerols (TGs) with different double bonds. Error bars indicate standard deviation. ‘*’ for *p* < 0.05 and ‘**’ for *p* < 0.01. If there is no mark between the columns, it indicates *p* > 0.05.

Another interesting lipid class was TGs, which did not show a change in the relative levels between the two mutants and BAA894. However, some unsaturated TGs, especially the polyunsaturated TGs with double bonds at 5, 7, 12, and 14, exhibited significant variation between the wild type and the mutants (Figure 6). Detailed information is provided as follows.

**Figure 7:**
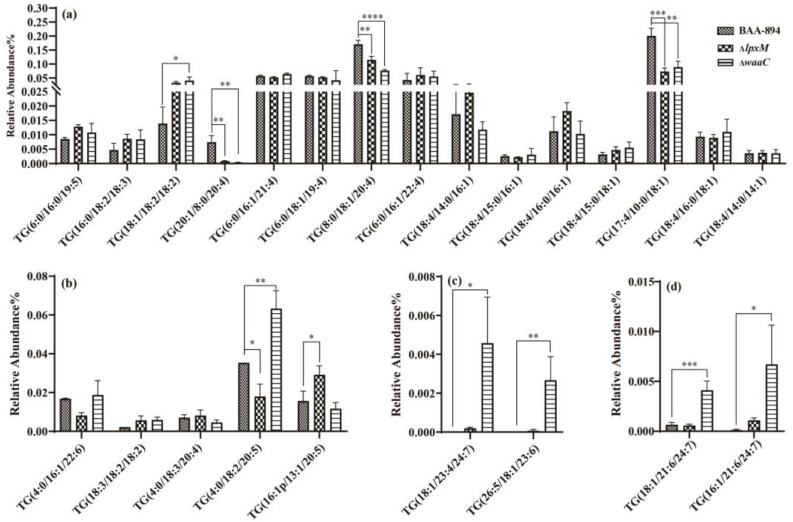
Abundance of unsaturated triacylglycerols (TGs) with 5 (**a**), 7 (**b**), 12 (**c**), and 14 (**d**) double bonds, respectively. Error bars indicate standard deviation. ‘*’ for *p* < 0.05, ‘**’ for *p* < 0.01, ‘***’ for *p* < 0.001, and ‘****’ for *p* < 0.0001. If there is no mark between the columns, it indicates *p* > 0.05.

As shown in Figure 7, among the unsaturated TGs with five double bonds (Figure 7a), TG(20:1/8:0/20:4), TG(8:0/18:1/20:4), and TG(17:4/10:0/18:1) were decreased in both mutants, while TG(18:1/18:2/18:2) was increased in Δ*waaC*. Other unsaturated TGs that exhibited changes included TG(4:0/18:2/20:5), TG(16:1p/13:1/20:5), TG(18:1/23:4/24:7), TG(26:5/18:1/23:6), TG(18:1/21:6/24:7), and TG(16:1/21:6/24:7). In previous reports, LPSs have been shown to activate triglyceride (TG) accumulation in phagocytes, suggesting a potential link between LPSs and TG synthesis [26]. The high variability of certain polyunsaturated TGs in Δ*lpxM* and Δ*waaC* provides new evidence that deserves further research.

## 4. Discussion

In the current study, for the first time, two mutants of *C. sakazakii* BAA-894 (Δ*lpxM* and Δ*waaC*), each with distinct alterations in the LPS structure, were used to evaluate their impact on resistance to environmental stresses encountered in dairy processing. Compared to BAA894, Δ*lpxM* and/or Δ*waaC* showed changed sensitivities to acidic, alkali, oxidative, osmotic, and desiccation stresses, indicating that LPSs play a critical role in the stress resistance of *C. sakazakii*. In most cases, the truncation of the inner core polysaccharide (Δ*waaC*) and/or the removal of the deacyl of lipid A (Δ*lpxM*) in LPSs impaired stress resistance. Peng Ma et al. investigated the membrane permeability of nine LPS structural mutants and found that any changes in the number and position of fatty acid chains on lipid A could increase the permeability of *Escherichia coli* cells. Among the factors affecting permeability, the chain length of LPS polysaccharides had the greatest impact, followed by phosphate groups and fatty acid chains [27]. In *Rhizobium leguminosarum*, mutations in *acpXL* and *lpxXL*, which eliminate the very long-chain fatty acids (VLCFAs) attached to lipid A, were found to increase the strain’s sensitivity to sodium chloride in 0.5% NaCl medium [28]. However, Δ*lpxM* displayed a significantly higher resistance to osmotic stress (Figure 1d), as *lpxM* can transfer the C14:0 secondary acyl chain to the 3′-position of Cronobacter sakazakii lipid A [10], suggesting the deficiency of one acyl chain in lipid A could enhance *C. sakazakii* resistance to osmotic stress. Given that the addition or removal of acyl chain(s) in lipid A frequently occurred in Gram-negative bacteria, it is crucial to investigate the correlation between lipid A modification and stress resistance in a broader range of *C. sakazakii* isolates.

It should be noted that Δ*waaC* displayed a significantly higher level of PIP(34:1/16:1), PIP(32:2/16:0), PIP(36:2/16:1), PIP(34:2/16:1), and PIP(36:5/16:0) compared to BAA894. PIPs are now attracting more and more interest in biochemistry, cell biology, and physiology, since they have diverse functions, such as involvement in cellular processes at the plasma membrane, including actin cytoskeleton dynamics, membrane dynamics, and ion channel regulation [29]. Additionally, they also work as signaling lipids involved in membrane association and protein orientation in human cancer development [30]. As far as we know, there were almost no reports on the specific role of PIPs in bacteria, and our findings provide valuable information that could inform future physiological studies on PIPs in bacterial systems.

The GP category exhibited the most diverse range of lipid species, with six of the sixteen classes (LPE, PC, CL, PI, PIP, and LdMePE) showing obvious changes in intensity compared to BAA894. As a dominant class with negatively charged polar head groups in bacterial membranes, PGs functioned as both membrane stabilizers and destabilizers, playing critical roles in controlling membrane/protein interactions [31]. In addition, PGs also participated in the sensing of extracellular stress and protein secretion across bacterial membranes. The roles of PGs in these processes are likely to become even more evident in the near future [32]. Therefore, further experiments are necessary to clarify the function of PGs in combating environmental stresses.

The present work demonstrated for the first time that the modifications in LPS structure affected bacterial resistance to environmental stresses. Simultaneously, the high diversity and variability observed in Δ*lpxM* and Δ*waaC* likely represent the biochemical foundation for their varied sensitivities to different stresses. Our findings provided valuable biochemical insights into the role of LPSs in *C. sakazakii* survival under stresses, and further investigations will be needed to understand the molecular mechanism in the future. The results of our work provide insights into the development of novel antimicrobial strategies that target LPS biosynthesis. By modulating the cell membrane lipid composition and disrupting the cell surface of *C. sakazakii* isolates with high resistance to environmental stresses, these strategies may offer a promising approach to combating this pathogen.

## Figures and Tables

**Table 1:** Bacterial strains used in this study.

Strains and Plasmids	Description	Source
Strains		
BAA-894	Wild-type *C. sakazakii*	ATCC
Δ*lpxM*	BAA-894 Δ*lpxM*	[10]
Δ*waaC*	BAA-894 Δ*waaC*	[7]

## Data Availability

The data and results of this study are available upon reasonable request. Please contact the main author of this publication.
